# Prevalence and risk factors of problematic internet use and the associated psychological distress among graduate students of Bangladesh

**DOI:** 10.1186/s40405-016-0020-1

**Published:** 2016-11-25

**Authors:** Md. Azharul Islam, Muhammad Zakir Hossin

**Affiliations:** 1 Department of Educational and Counselling Psychology, University of Dhaka, Dhaka, 1000 Bangladesh; 2 Department of Public Health Sciences, Karolinska Institute, Stockholm, Sweden

**Keywords:** Internet addiction test, General health questionnaires, Socioeconomic status, Young adults, Bangladesh

## Abstract

A growing body of epidemiological literature suggests that problematic Internet use (PIU) is associated with a range of psychological health problems in adolescents and young adults. This study aimed to explore socio-demographic and behavioural correlates of PIU and examine its association with psychological distress. A total of 573 graduate students from Dhaka University of Bangladesh responded to a self-administered questionnaire that included internet addiction test (IAT), 12-items General Health Questionnaire and a set of socio-demographic and behavioural factors. The study found that nearly 24% of the participants displayed PIU on the IAT scale. The prevalence of PIU significantly varied depending on gender, socioeconomic status, smoking habit and physical activity (p < 0.05). The multiple regression analyses suggested that PIU is strongly associated with psychological distress regardless of all other explanatory variables (adjusted OR 2.37, 95% CI 1.57, 3.58). Further research is warranted to confirm this association by employing prospective study designs.

## Background

Problematic Internet use (PIU) or Internet addiction (IA) has been extensively researched since the mid-1990s, particularly in some Western and Asian countries. Although considerable evidence shows that PIU is associated with a number of negative health outcomes in adolescents and adults (Ko et al. 2012; Kuss et al. 2013a), it was not officially classified as a clinical disorder in the latest edition of the *Diagnostic and Statistical Manual for Mental Disorders* (*DSM*-*V*) (APA 2013). This indicates the need for further evidence on this emerging mental health epidemic.

Examining the prevalence and factors of PIU and the associated health problems is particularly important in a country like Bangladesh where the growth of Internet use is faster than socio-economic development itself. In line with global technological development, the use of Internet has also accelerated in Bangladesh, with the number of Internet users increasing from 0.1 million in 2000 to 62 million in 2016 (BTRC 2016). This unprecedented growth of Internet may pose a variety of health challenges, if proper measures are not taken in the right time. Perhaps the most important reason is the unconditional and positive acceptance of its use among common users as well as policy makers. The proliferation of the use of Internet has been largely considered an indicator of development. The government gives tremendous effort to make this happen, that is, to provide each citizen access to Internet with the ultimate aim of building a ‘Digital Bangladesh’ by 2021. Therefore, it is plausible to assume that people as a whole are not aware of the problematic use of Internet and its effects on their psychological health.

PIU has been defined as ‘an individual’s inability to control their internet use, which in turn leads to feelings of distress and functional impairment of daily activities’ (Shapira et al. 2000). It is a condition akin to ‘impulse-control disorder’ that does not involve an intoxicant similar to symptoms of pathological gambling, overeating and so on (Young 2004). Kimberly S. Young was one of the key proponents of the construct ‘Internet Addiction’. She proposed eight diagnostic criteria adapted from the symptoms of pathological gambling (DSM-IV) for detecting an individual’s Internet use as problematic (Young 1996). These are cognitive preoccupation with the Internet, increased tolerance, unsuccessful attempts to decrease use, withdrawal symptoms, staying online much longer than needed, lying about online activity, negative emotion resulting from online activity, and use of the Internet to self-medicate. Clearly, an individual with these symptoms will face difficulty in normal daily functioning and may experience poor mental health. To test this hypothesis, it is crucial for professionals to measure PIU systematically. A number of tools have been developed out of which Young’s internet addiction test (IAT) was found to have wide uses (Young 1998). IAT gives a score ranging between 20 and 100, with a higher score indicating a greater degree of PIU.

Studies examining the prevalence of PIU lack consensus on the diagnostic criteria set to identify PIU due to various measures and cut off points used (Kuss et al. 2013a, b). Considering only Young’s IAT with a cut-off point of 70 and above, a recent meta-analysis of 164 prevalent figures from 31 nations across seven world regions estimated a global prevalence of IA as 6.0% (95% CI 5.1–6.9) (Cheng and Li 2014). This classification of PIU, however, did not consider moderate users who are also unable to control their Internet use (Young 2016). Therefore, incorporating moderate and excessive users as defined by IAT would be safe to detect PIU (i.e., PIU = IAT score 50+). Following this notion, the prevalence of PIU appears to be high. For instance, a recent epidemiological study conducted in six Asian countries revealed the prevalence of PIU as follows: Philippines (51%), Japan (48%), China (19%), Hong Kong (35%), South Korea (14%), and Malaysia (37.5%) (Mak et al. 2014). No such comprehensive investigations have been carried out in Bangladesh, though a preliminary assessment with a small sample of university students (n = 172) found that 36% of the participants scored equivalent to PIU (Nigar and Karim 2014).

The majority of the studies, however, reported the prevalence, predictors and consequences of addictive use of Internet using adolescent samples (e.g., Kuss et al. 2013b; Lam et al. 2009; Liberatore et al. 2011; Yu and Shek 2013). Much less is known about the young adults, notably the university graduates, who are at particular risk of PIU due to several reasons. Firstly, they have comparatively wide and easy access to the Internet via campus laboratory, free campus Wifi Zone and cheap mobile Internet packages. This explains, in part, why 90% of the social media users in the USA belong to the age group 18–29 (Perrin 2015). In Bangladesh, 94% of the total subscribers accessed Internet via mobile phones until April 2016 (BTRC 2016) and obviously, youths are the major consumers of the mobile Internets. Secondly, the youth, irrespective of their living arrangement such as living with family or in university dormitories, typically enjoy the freedom of their choices and Internet use. Next, considering their developmental period in which they are striving to build own identity, career and partner, they may use Internet to facilitate the expected growth, which in turn may appear as an addictive behaviour (Lanthier and Windham 2004). According to Wallace, ‘there is no question that twernty-first century youth have become far more dependent upon connectivity for studying, playing, communicating, and socializing’ (Wallace 2014). Existing literature also demonstrates a prevalence of PIU among university/college students from 0.8% in Italy (Poli and Agrimi 2012) to a moderate level of 18.3% in UK (Niemz et al. 2005) to a higher level of 40% in Jordan (Al-Gamal et al. 2015).

PIU has been found to be associated with a broad range of negative health outcomes with some exceptions. For example, increased depressive symptoms were associated with spending increased time in online activities namely shopping and gambling but inversely with chatting, communication and email (Morgan and Cotten 2003). That is, spending time on social networking was positively associated with mental health. However, recent evidence shows an overall negative relation with online networking and well-being (Sabatini and Sarracino 2014) which indicates that the normal use of Internet could turn into problematic over time. In addition to well-being, PIU was also linked with adolescents’ psychosomatic (i.e., poor physical energy, physiological dysfunction, and poorer immunity), behavioural and emotional symptoms (Cao et al. 2011). Adolescents with PIU are also at risk of relatively high depression and poor social adaptation. Lam and Peng (2010), for example, prospectively examined the effect of pathological Internet use and demonstrated that the adolescents with addictive use of Internet were at a relatively high risk of developing depression in the follow up. Although Internet provides an avenue for easy social interactions, transferring these skills into real life is difficult, as it requires exposures to real life situations. Furthermore, psychiatric co-morbidity of IA has also been well-documented particularly for substance use disorder, attention-deficit hyperactivity disorder (ADHD), depression, hostility, and social anxiety disorder [see Ko et al. (2012) for a review].

Specific studies on PIU and psychological well-being among university students were limited to explaining simple association of these two constructs. For instance, PIU was positively correlated with psychological distress as measured by perceived stress scale (Cohen et al. 1983) in a sample of university students in Jordan (Al-Gamal et al. 2015). Niemz et al.(2005) assessed psychological distress by the 12-items General Health Questionnaire (GHQ-12) and PIU of 371 British students and found that there was no significant difference in the mean score of GHQ-12 among PIU and non-PIU groups [F(2, 368) = 2.15; p = 0.118].

While there is a plethora of research focusing on a general negative association of PIU with psychological well-being, very little is known about the role of the socio-demographic determinants or the lifestyle factors in the association of interest. For example, socio-demographic factors such as age, sex, socio-economic status (SES), relationship status, living arrangements as well as behavioural factors such as smoking, physical activity and sleeping habit are generally found to be associated with overall psychological well-being (Bayram and Bilgel 2008; Glozier et al. 2010; Matsuzaki et al. 2007). Short habitual sleep duration, for instance, has been shown to be linearly associated with persistent psychological distress in young adults (Glozier et al. 2010). Again, a breakup in a committed relationship usually results in symptoms of depression, loneliness and even self-harm tendencies (Rhoades et al. 2011). Furthermore, the lower the SES, the poorer the overall health, popularly known as the social gradient in health (Marmot 2009). In a similar vein, males generally report a higher prevalence of PIU than females (Ha and Hwang 2014; Shahnaz and Karim 2014). These factors, in turn, could play a crucial role in the association between PIU and psychological distress. Therefore, an individual with lower psychological well-being could be the victim of many other factors and not necessarily due to his or her problematic use of Internet. Consequently, an investigation into the association between PIU and psychological well-being with a simultaneous focus on the relevant socio-demographic and lifestyle factors would be a useful contribution.

To our knowledge, only three studies with comparatively small sample sizes focused on IA in Bangladesh (Karim and Nigar 2014; Nigar and Karim 2014; Shahnaz and Karim 2014) but none investigated IA in relation to psychological distress or its socio-demographic and behavioural determinants. The present paper, therefore, is the first of its kind in the context of Bangladeshi society. The paper aims to report the prevalence and risk factors of PIU among young Bangladeshi adults and examine the association between PIU and psychological distress with a particular attention paid to the role of socio-demographic and health behavioural factors in the association of interest. Specifically, the paper addresses the following research questions: (1) What is the prevalence of PIU among the graduate students of Bangladesh? (2) Does the prevalence of PIU differ significantly between different social groups? (3) Is PIU associated with psychological distress independent of the sociodemographic and behavioural factors? (4) To what extent is the association between PIU and psychological distress explained by the socio-demographic and behavioural factors?

## Methods

### Participants

The participants of this study were the graduate students of Dhaka University (DU) of Bangladesh. DU is one of the oldest universities in the South Asian region, established in 1921. Currently, there are around 37,000 students pursuing their higher studies in 77 different departments, categorized under 13 faculties and 11 institutes. Studying at DU is almost free, as the state bears almost the entire tuition. Therefore, students from all socioeconomic backgrounds can pursue higher studies once they pass the very competitive entrance exam. We chose DU for this study due to its diverse socio-economic characteristics of the students. Around 600 graduates of DU from various faculties were approached for data collection, during January and February 2015. Out of them, data from 573 participants were kept for final analyses as a few of them refused to take part and some returned the incomplete questionnaires. Participants were selected based on a stratified random sampling design and the sample was representative of all faculties. A detailed description of the study sample can be found in Table 1.

**Table 1:** Sample distribution and the prevalence of problematic Internet use by socio-demographic and behavioural factors

Characteristics	Sample	Problematic Internet use		p-for-difference
		Yes	No	
	% (n)	% (n)	% (n)	
Total	100 (573)	23.9 (137)	76.1 (436)	
Age (years)				0.520
20–25	60.7 (348)	23.0 (80)	77.0 (268)	
26–30	39.3 (225)	25.3 (57)	74.7 (168)	
Sex				<0.001
Female	30.2 (173)	12.7 (22)	87.3 (151)	
Male	69.8 (400)	28.8 (115)	71.2 (285)	
Socioeconomic status				<0.05*
High	22.7 (130)	19.2 (25)	80.8 (105)	
Middle	62.0 (355)	22.8 (81)	77.2 (274)	
Low	15.4 (88)	35.2 (31)	64.8 (57)	
Relationship status				0.071
Single	42.6 (244)	24.6 (60)	75.4 (184)	
Partnered	40.3 (231)	19.9 (46)	80.1 (185)	
Separated	17.1 (98)	31.6 (31)	68.4 (67)	
Living arrangement				0.930
With family	29.5 (169)	23.7 (40)	76.3 (129)	
Dorm/mess	70.5 (404)	24.0 (97)	76.0 (307)	
Smoking				<0.05
No	80.1 (459)	21.8 (100)	78.2 (359)	
Yes	19.9 (114)	32.5 (37)	67.5 (77)	
Physical activity				<0.05
No	49.4 (283)	27.9 (79)	72.1 (204)	
Yes	50.6 (290)	20.0 (58)	80.0 (232)	
Sleep duration				0.693
Normal sleep	66.1 (379)	23.0 (87)	77.0 (292)	
Short sleep	29.7 (170)	25.3 (43)	74.7 (127)	
Long sleep	4.2 (24)	29.2 (7)	70.8 (17)	

* Instead of p-for-difference, p-for-trend has been reported for socioeconomic status

### Procedure

A written informed consent was obtained from each participant before collecting the data. Participation in the study was voluntary. Participants were not given any privilege or not discriminated in any means due to their participation or not participation. Prior to commencing the study, ethical approval was taken from the concerned university ethics committee. Five research assistants holding at least a Master degree in psychology/clinical/counselling psychology were employed for data collection. They were given necessary training on the research tool, data collection procedure and ethical issues of research.

### Measures

#### Psychological distress

The main dependent variable in the study is psychological distress. The Bangla validated (Sorcar and Rahman 1990) 12-item version of General Health Questionnaire (Goldberg 1972) (GHQ-12) was used to measure psychological distress. This is one of the widely used self-reported measures of general psychological health. This instrument was originally developed as a screening test for detecting minor psychiatric disturbance or strain. The measure assesses changes in affective and somatic symptoms relative to usual levels of health, e.g., feelings of strain, depression, inability to cope, anxiety-based insomnia and lack of confidence (Goodchild and Duncan-Jones 1985). There are four different scoring procedures available for GHQ-12 (Goldberg et al. 1998). In this study, we followed the 0–0–1–1 scoring procedure which produces a score ranging from 0 to 12, with a higher score indicating higher psychological distress. Again, there are various thresholds for detecting psychological distressed. For a safe detection, the mean score has been suggested as a potential threshold (Goldberg et al. 1998). The mean GHQ-12 score for this study was 3.03. Therefore, we chose ‘three’ as the cut-off point for detecting psychologically distressed cases. Internal consistency of the Bangla GHQ-12 for this sample was very good (Chornbach’s α = 0.85).

#### PIU

The main independent variable used in the study is PIU which was assessed by Young’s IAT (Young 1996), the first psychometrically valid tool to measure problematic use of Internet. This 20-item scale is designed to measure psychological dependence, compulsive use, and withdrawal as well as related problems of school, sleep, family, and time management. Every item is scored on a five-point Likert scale ranging from 1 (rarely) to 5 (always). Total score is the sum of all items giving a range of 20 to 100 where a higher score indicates greater level of IA. For this study the Bangla validated IAT (Karim and Nigar 2014) was used. The Bangla version of IAT retained 18 items instead of original 20 items with a four factor structure. The score of Bangla IAT, therefore, ranges between 18 and 90. There is no gold standard for distinguishing between PIU and non-PIU (Kuss et al. 2013a, b) with cut points varying from 50 (Kormas et al. 2011) to 80 (Liberatore et al. 2011; Ostovar et al. 2016). According to the IAT manual, users are given different labels based on the total score obtained on the IAT scale. These are: normal user (IAT total ≤30), mild user (IAT total = 31–49), moderate user (IAT total = 50–79) and severe or excessive user (IAT total ≥80) (Young 2016). Since the moderate users are often unable to control their Internet use (Young 2016), we considered both moderate and excessive use of Internet as problematic. This notion of PIU classification is fairly supported by the existing literature (Al-Gamal et al. 2015; Ghamari et al. 2011; Kormas et al. 2011; Lam and Peng 2010; Ni et al. 2009). Since the Bangla IAT score ranges from 18 to 90, a score of 45 or above has been defined as PIU in the present study. The tool has been tested in different studies with sound psychometric properties (Jelenchick et al. 2012; Widyanto and McMurran 2004). The Bangla IAT and its factors have been found to have sound reliability and strong convergent and discriminant validity (Karim and Nigar 2014). Internal consistency for this sample was found excellent (α = 0.91).

#### Socio-demographic and behavioural measures

Demographic information consisting of age, sex, living arrangement (with family vs dorm/mess), relationship status (single, partnered, separated) and perceived SES were recorded through a separate demographic information recording sheet. SES was measured by asking the respondents to think about their society and respond where they belong to in terms of education, money and job: upper class, middle class or lower class. The health behavioural factors included in the study were physical activity, sleep duration and smoking. To assess physical activity, respondents were asked if they participate in moderate physical activities for at least 10 min at a time each day, such as brisk walking, cycling, swimming or any other activity that causes some increase in breathing or heart rate. As for sleep duration, respondents’ were asked to report their average duration of sleep per day. Based on the US National Sleep Foundation’s recommendations, sleep duration was categorized as normal (7–9 h), short (<7 h) and long sleep (>9 h) (Hirshkowitz et al. 2015). Participants also reported whether they were smoking cigarette by answering ‘yes’ or ‘no’.

### Statistical analyses

All data were analysed using the Statistical Package for Social Sciences (SPSS) version 20. Unadjusted prevalence percentages of PIU were obtained for the total sample and for all variables of interest. Chi square test for independence was used to assess the differences in the prevalence of PIU by socio-demographic and behavioural factors. The associations between PIU, other explanatory variables and psychological distress were examined at both bivariate and multivariate levels, using binary logistic regression. The regression analyses were based on three models. The unadjusted odds ratios (OR) for the associations between all explanatory variables and psychological distress were estimated in model 1. Model 2 explored the association between PIU and psychological distress, controlling for the socio-demographic variables. Model 3 represents the fully adjusted model that further controlled for the behavioural factors. All point estimates were reported with 95% confidence intervals.

## Results

Table 1 shows the distribution of the sample as well as the prevalence of PIU by socio-demographic and behavioural factors. Nearly 24% of the total sample falls under the category of PIU. The age of the study participants ranges from 20 to 30 with a mean of 25.1. Almost two-thirds of the participants were males (69.8%) out of which 28.8% were classified as problematic Internet users. The proportion of PIU between male and female differs significantly (p < 0.001). Regarding SES, most of the participants reported to be in the middle class (60.0%), a few were in low SES (15.4%) and the others belonged to high SES (22.7%). The prevalence rates of PIU across SES categories indicate a negative trend which means that the higher the SES, the lower the prevalence of PIU. This trend is statistically significant at <0.05 level (p-for-trend: 0.018). Around 40% of the participants were in partnered (romantic) relationship while 42.6% were single. A considerable proportion of the participants (17.1%) also reported to have split up their romantic relationship. In general, the prevalence of PIU is more common in this group compared to those who are single or partnered but the differences between them are statistically significant only at <0.1 level. Around 70% of the study participants reported to be living in either university or private dormitories while the remaining was living with their families. The proportion of PIU was mostly similar across the living status of the participants. As regards the behavioural factors, PIU was higher among smokers compared to non-smokers (32.5 vs 21.8%). The difference of the proportion of the number of smokers and non-smokers by PIU status was statistically significant (p < 0.05). Compared to those engaged in moderate or vigorous physical activity, the prevalence of PIU was also significantly higher for those not engaged in physical activity (20.0 vs 27.9%). The duration of sleep of the study participants, however, did not show any statistical significance in terms of the prevalence of PIU.

The prevalence of psychological distress among the study participants was 28.4% (data not shown). The associations of psychological distress with PIU and other socio-demographic and behavioural correlates are presented in Table 2. The crude ORs from the logistic regression analyses demonstrate that PIU is associated with a 2.63-fold increased odds of having psychological distress. When the socio-demographic factors were entered in the model, the effect size became slightly smaller but still remained significant and robust (OR 2.48, 95% CI 1.65, 3.72). Further statistical adjustment for the lifestyle factors did not attenuate the association considerably. As the multivariate analysis in model 3 suggests, PIU is strongly associated with a higher prevalence of psychological distress (OR 2.37, 95% CI 1.57, 3.58) even when all other factors are held constant. Only a small part (<10%) of the original association was accounted for by the socio-demographic and behavioural factors considered in the analyses. However, of all the explanatory variables included in the fully adjusted model, low SES emerged as the strongest predictor of psychological distress, followed by PIU and smoking. The study results further show that there is a clear socioeconomic gradient in psychological distress, with the individuals at the bottom of the socioeconomic hierarchy reporting more psychological problems than those at the top. For example, the individuals belonging to the low socioeconomic class has 2.45 times elevated odds (95% CI 1.33, 4.50) of psychological distress relative to the high socioeconomic class. The corresponding odds of psychological distress among those identifying themselves as middle class is 1.62 (95% CI 1.01, 2.61). Furthermore, the estimated odds of reporting psychological distress is significantly higher among the smokers (OR 1.86, 95% CI 1.17, 2.96) compared to the non-smokers. No statistically significant interactions, however, were found between PIU and other covariates in regard to the prevalence of psychological distress.

**Table 2:** Association between problematic Internet use and psychological distress, controlling for socio-demographic and behavioural correlates (n = 573)

Explanatory variables	Model 1	Model 2	Model 3
	OR [95% CI]	OR [95% CI]	OR [95% CI]
Problematic Internet use			
No [ref.]	1.00	1.00	1.00
Yes	*2.63* [1.77, 3.90]	*2.48* [1.65, 3.72]	*2.37* [1.57, 3.58]
Age (years)			
20–25 [ref.]	1.00	1.00	1.00
26–30	0.84 [0.59, 1.20]	0.79 [0.55, 1.15]	0.78 [0.53, 1.14]
Sex			
Female [ref.]	1.00	1.00	1.00
Male	1.44 [0.98, 2.12]	1.14 [0.74, 1.74]	0.99 [0.63, 1.55]
Socioeconomic status			
High [ref.]	1.00	1.00	1.00
Middle	*1.60* [1.02, 2.53]	*1.59* [1.00, 2.54]	*1.62* [1.01, 2.61]
Low	*2.55* [1.43, 4.55]	*2.21* [1.22, 4.02]	*2.45* [1.33, 4.50]
Relationship status			
Single [ref.]	1.00	1.00	1.00
Partnered	1.02 [0.70, 1.50]	1.07 [0.72, 1.60]	1.02 [0.68, 1.54]
Separated	1.24 [0.76, 2.03]	1.16 [0.70, 1.93]	1.07 [0.64, 1.80]
Living arrangement			
With family [ref.]	1.00	1.00	1.00
Dorm/mess	1.22 [0.83, 1.79]	1.22 [0.81, 1.84]	1.25 [0.83, 1.89]
Smoking			
No [ref.]	1.00		1.00
Yes	*1.87* [1.23, 2.84]		*1.86* [1.17, 2.96]
Physical activity			
No [ref.]	1.00		1.00
Yes	.85 [0.58, 1.20]		0.84 [0.58, 1.21]
Sleep duration			
Normal sleep [ref.]	1.00		1.00
Short sleep	0.81 [0.55, 1.20]		0.84 [0.56, 1.26]
Long sleep	1.89 [0.83, 4.33]		1.88 [0.79, 4.48]

The ORs in italics indicate statistical significance

Model 1: unadjusted

Model 2: adjusted for age, sex, socioeconomic status, relationship status, and living arrangement

Model 3: adjusted for model 2 + smoking, physical activity, and sleep duration

*OR* odds ratio,* CI* confidence interval

## Discussion

The study sought to report the prevalence of PIU among graduate students of Bangladesh and its distribution by socio-economic and behavioural characteristics of the participants. In addition, the study explored the association between PIU and psychological distress controlling for the socio-demographic and behavioural covariates. Results revealed that nearly 24% of the participants used Internet problematically. There was a significant sex difference in the prevalence of PIU with males reporting PIU more than females. The prevalence rates of PIU also varied across socioeconomic status, relationship status, smoking habit and physical activity. The study found that a strong and robust association of PIU with psychological distress persists even when the socio-demographic and behavioural factors are adjusted for.

The prevalence of PIU found in this study (23.9%) is lower than the rates obtained in university samples of similar age groups from Muslim majority countries like Jordan (40%) and Iran (39.6%) but higher than the British sample (18.3%) (Al-Gamal et al. 2015; Ataee et al. 2014; Niemz et al. 2005). When both moderate and excessive users of Internet are incorporated into PIU according to the IAT, the prevalence rates are usually higher. The prevalence figures from Asian countries affirms this assertion (Mak et al. 2014). Furthermore, time could be another potential determinant of high prevalence. With time, the use of Internet increases, so does the PIU. Evidence from earlier studies tended to indicate a lower prevalence than the most recent studies. Nonetheless, the prevalence of PIU found for the Bangladeshi sample bears potential to draw clinical attention as roughly one out of four students is problematic Internet user.

In agreement with previous literature, this study shows that males were more predisposed to PIU behaviour (Ha and Hwang 2014; Morahan-Martin and Schumacher 2000; Shahnaz and Karim 2014). The sex difference in PIU is commonly explained by the distinct personality patterns of males and females and the purpose of using Internet. Males, for instance, are usually more enthusiastic about exploring the unknown or discovering new inventions (e.g., high on sensation seeking) (Ball et al. 1984). This, in turn, could gradually lead to compulsive Internet use as Internet is currently the main hub of all the exciting adventures. Secondly, males are found to engage more in addictive contents such online gaming, pornography and cybersex compared to their female counterparts (Bruno et al. 2014; Doornwaard et al. 2016; Young 1999). It was found that males score low particularly in the time control factor of IAT, indicating a lack of control over time when using Internet, a leading cause of PIU (Bruno et al. 2014).

SES is another strong risk factor of PIU. In this study, SES has been inversely associated with PIU, that is, the lower the SES, the higher the PIU. This finding is also in line with the evidence found in a Greek study which demonstrated that lower socioeconomic position was associated with higher score on the ‘neglect of social life’ factor of IAT (Andreou and Svoli 2013). Generally, the students from low socioeconomic backgrounds migrate to the capital city from the rural parts of the country to study at the premier public university, leaving behind their close relatives. They are, therefore, more likely to consider online engagement as a strategy to alleviate their social isolation and loneliness. This online engagement, if not controlled, could appear as problematic (Caplan 2007; Kim and Haridakis 2009). PIU was also found to be more common among those who had a ‘breakup’ in their romantic relationship. Existing literature suggests that split-up romantic relationship may cause poor mental health (Monroe et al. 1999; Simon and Barrett 2010) by increasing frustration and social anxiety which, as a consequence, might manifest as addictive behaviours like PIU (Liu and Kuo 2007). We also found that PIU was higher among the participants who smoke cigarette and those not engaged in physical activity. An earlier study focusing on an adolescent sample in China also found non-involvement in physical activity as a risk factor for PIU, although smoking was not significantly associated with IA (Lam et al. 2009). Based on a nationally representative sample, Lee et al. (2013), however, found that smoking predisposes to a high risk of IA (OR 1.203, p = 0.004) even after adjusting for sex, age, stress, depressed mood and suicidal ideation. It is possible that smoking and IA share similar bio-psycho-social mechanisms—a potential area for further investigation.

The study results further demonstrate that PIU is significantly associated with psychological distress, a finding which is largely in agreement with previous studies (Al-Gamal et al. 2015; Cotten et al. 2011; Ha and Hwang 2014; Shapira et al. 2000; Tsai et al. 2009). We, however, anticipated that this association would be substantially attenuated once the socio-demographic and behavioural factors are adjusted for, given the well-established links between these factors and psychological wellbeing (Bayram and Bilgel 2008; Glozier et al. 2010; Matsuzaki et al. 2007). To our surprise, only an insignificant part of the observed association was explained by these factors, despite the fact that SES and smoking appeared as strong predictors of psychological distress in the fully adjusted regression model. There could be several possible explanations for that. One crucial feature of PIU is the lack of control over the use of Internet. Thus, one may end up in distress after spending increasing amount of time online. The individual may promise not does it again but later get engaged in the same addictive behaviour, leading to more frustration and distress. It thus goes like a vicious cycle. The psychological distress, therefore, might arise due to the negative consequences this cycle brings on the academic performance (Jiang 2014), unhealthy lifestyle such as irregular meal and bedtime (Nigar and Karim 2014) and interpersonal relationships (Cao et al. 2011). Moreover, in the case of adolescents, a common interpretation of PIU is that the problematic users use Internet as a way of escaping from the real life stress or challenges (Kim and Davis 2009). There is a high possibility that graduate students also over engage themselves in online activities in order to cope with the strain associated with their career choice—a common stressor during this period. This is the time when the graduates struggle to shift to working life from student life. Since the unemployment rate is high in Bangladesh, many of them remain uncertain about their future career position. Responding to real life situation in this way, i.e., by over engaging online, however, could manifest as poor psychological health such as distress. Because PIU is kind of maladaptive coping strategy that limits or cuts down on actual physical activity or involvement, both are crucial for psychological well-being. Furthermore, PIU co-morbids with other mental health conditions such as ADHD, social anxiety, phobia, depression, loneliness and low self-esteem (Cao et al. 2011; Liberatore et al. 2011). The simultaneous presence of two or more health conditions can influence one another in terms of symptoms moderation. As mentioned earlier, psychological distress in this study has been conceptualized as changes in affective and somatic symptoms relative to usual levels of health and hence PIU might affect other possible co-morbid conditions which potentially result in psychological distress within the individual. A closer look at these issues could unearth the underlying possible mechanism of this association.

The findings of our study, however, have to be cautiously interpreted as it has some limitations. Firstly, due to the nature of cross-sectional design, we cannot infer a causal connection between PIU and psychological distress. That is, the link between PIU and psychological distress as observed in this study is simply associational and hence we could not rule out the possibility that psychological distress causes PIU rather than the other way round. A longitudinal study design would be more appropriate to unravel the possible causal direction of the association. Secondly, the study sample was derived from one university only. Although, the sampled university is the largest populated academia in the country, incorporating participants from other universities could more accurately reflect the scenario under investigation. Thirdly, as indicated above, we did not consider some potential confounding factors such as the participants’ academic pressure, the pressure to find a job, the purpose of using Internet, loneliness and comorbid mental health problems which are likely to bias the association of interest.

Despite these limitations, the study findings carry important implications for planning public health policies and interventions. Our study findings, for example, imply that the males, low socioeconomic groups, smokers and the physically inactive young population can be the important target groups for any intervention designed to prevent problematic use of Internet. Besides, the findings of this study can aid the mental health service providers in their clinical practices, especially when it comes to dealing with the patients with IA behaviour. Regardless of the causal direction of the association between PIU and psychological distress, the mental health professionals such as university counsellors or psychotherapists are expected to pay attention to the signs and symptoms of PIU during the assessment and formulation of psychological health issues of the patients as PIU is strongly associated with their psychological wellbeing. However, further research is warranted for a broader and more in-depth understanding of the link between PIU and psychological distress taking into account the comorbid mental health conditions, academic performance, psycho-social stressors and interpersonal problems of the young adults of Bangladesh.
